# The microstrain-accompanied structural phase transition from h-MoO_3_ to α-MoO_3_ investigated by in situ X-ray diffraction

**DOI:** 10.3762/bjnano.14.55

**Published:** 2023-06-07

**Authors:** Zeqian Zhang, Honglong Shi, Boxiang Zhuang, Minting Luo, Zhenfei Hu

**Affiliations:** 1 School of Science, Minzu University of China, 27 Zhong guancun South Avenue, Haidian District, Beijing, 100081, China; 2 Institutional Center for Shared Technologies and Facilities of Institute of Process Engineering, Chinese Academy of Sciences, Beijing, 100190, China

**Keywords:** microstrain, molybdenum oxide, phase transition, thermal expansion

## Abstract

In situ X-ray diffraction indicates that the structural phase transition from h-MoO_3_ to α-MoO_3_ is a first-order transition with a phase transition temperature range of 378.5–443.1 °C. The linear coefficients of thermal expansion of h-MoO_3_ are strongly anisotropic, that is, α*_a_*_=_*_b_* = 72.87 × 10^−6^ K^−1^ and α*_c_* = −19.44 × 10^−6^ K^−1^. In the h-MoO_3_ phase, water molecules are located at the (0 0 0.25) site inside the MoO_6_ octahedra tunnel that is formed by six MoO_6_ corner-sharing octahedron zigzag chains. With increasing temperature, the release of water molecules from the octahedra tunnel causes the octahedra chains to shrink and the octahedra tunnel to expand. When the phase transition occurs, the anomalous expansion of the MoO_6_ octahedra tunnel ruptures the Mo–O_2_ bonds, forming individual MoO_6_ octahedron zigzag chains that then share corners to generate octahedron layers in the ⟨100⟩_α_ direction. The octahedron layers are bonded by van der Waals interactions in the ⟨010⟩_α_ direction, crystalizing into the α-MoO_3_ structure.

## Introduction

Molybdenum exhibits oxidation states ranging from +2 to +6 [1–2], leading to a range of molybdenum oxides. Molybdenum oxides include the fully stoichiometric MoO_3_ with a large bandgap above 2.7 eV, the reduced oxides MoO_3−_*_x_* with oxygen vacancies, and the semimetal MoO_2_. The degree of reduction influences the bandgap energy of molybdenum oxides, making them multifunctional electronic and optical materials for applications in ion batteries [3–4], lubricants [5], gas detectors [6–7], photochromism [8–9], photocatalysis [10–11], and superconductors [12–13].

The molybdenum oxide MoO_3_ can crystalize into several structures, including α-MoO_3_ [14], β-MoO_3_ [15–16], h-MoO_3_ [17], γ-MoO_3_ [18], and the high-pressure phase MoO_3_-II [19]. α-MoO_3_ and β-MoO_3_ are the two most commonly reported molybdenum oxides. α-MoO_3_ is a thermodynamically stable orthorhombic phase. It is a layered crystal with strong covalent bonding within the layers and weak van der Waals coupling between layers [20]. β-MoO_3_ is a metastable phase in which the MoO_6_ octahedra share corners in three dimensions to construct a monoclinic structure [16].

h-MoO_3_ is a metastable hexagonal phase. It has the unique structural characteristic that the MoO_6_ octahedra chains share corners to form large one-dimensional tunnels with a diameter of ca. 3 Å [21]. The tunnel permits the intercalation of cations, water molecules, and ammonia, which leads to better photophysical and photochemical capabilities of h-MoO_3_ compared to the other MoO_3_ structures [17,22]. However, there are still some question regarding the h-MoO_3_ phase: (1) The location of the intercalated molecules inside the h-MoO_3_ structure is unknown. (2) The reason of the mismatch in h-MoO_3_ thin films and the failure of microdevices is not understood. (3) The structural phase transition at the atomic scale from h-MoO_3_ to α-MoO_3_ is still unclear.

Here, to reveal the features of the structural phase transition from h-MoO_3_ to α-MoO_3_, we performed in situ X-ray diffraction experiments at temperatures ranging from 30 to 450 °C. The Rietveld refinement results indicate water molecules at the (0 0 0.25) site inside the MoO_6_ octahedra tunnel. Before the phase transition, the release of the water molecules causes the octahedra chains to shrink and the octahedra tunnel to expand, which results in a strongly anisotropic thermal expansion. When the phase transition occurs, the anomalous expansion of the MoO_6_ octahedra tunnel ruptures the Mo–O_2_ bonds, forming individual MoO_6_ octahedron zigzag chains that share corners to generate octahedron layers. The octahedron layers are bonded by van der Waals interaction, crystalizing into the α-MoO_3_ structure.

## Results and Discussion

### Features of the phase transition from h-MoO_3_ to α-MoO_3_

**Figure 1:**
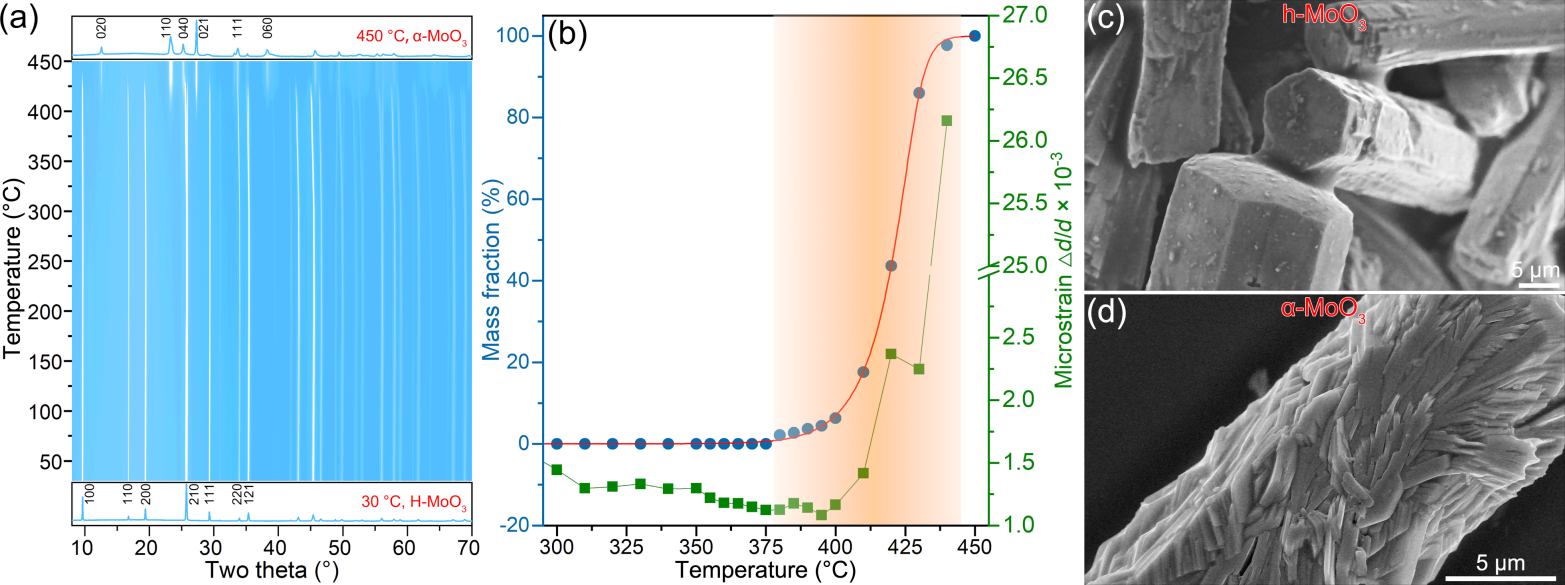
(a) 3D plot of the in situ X-ray diffraction patterns, where bottom and top display the two patterns collected at 30 and 450 °C, respectively. (b) Mass fraction of the α-MoO_3_ phase and the microstrain in the h-MoO_3_ phase. (c,d) SEM microstructures of samples calcinated at 300 and 450 °C, respectively.

To observe the crystal structure evolution of h-MoO_3_ induced by temperature, a thoroughly powdered sample was used to perform in situ X-ray diffraction measurements during heating from 30 to 450 °C, as shown in Figure 1a. At 30 °C, all diffraction peaks can be well indexed to the hexagonal phase h-MoO_3_. The space group of h-MoO_3_ is *P*6_3_/*m*, and the refined lattice parameters are 10.5629(4) × 3.7260(1) Å. (Note that the PDF card with No. 21-0569 overestimates the unit cell (10.531 × 14.876 Å) and the number of formula units (*Z* = 51), although this card can be matched with the experimental pattern.) The intensity of the measured (100) peak is obviously weaker than that of the standard peak, but the height of the (210) peak is noticeably stronger. This indicates the presence of a strong preferred orientation in the sample, although it has been properly powdered. When the temperature was raised to 380 °C, some weak diffraction peaks at 12.56°, 23.29°, 25.25°, and 27.29° appeared, indicating the beginning formation of a new phase. At 450 °C, the hexagonal phase disappeared entirely, and the XRD pattern became that of the orthorhombic phase (PDF#35-0609). For the orthorhombic phase, α-MoO_3_, the space group is *Pnma*, and the refined lattice parameters are 3.9804(4) × 14.1545(1) × 3.6967(2) Å.

To determine the transition temperature of the h-MoO_3_→α-MoO_3_ phase transition, the mass fraction of α-MoO_3_ was determined via Rietveld refinement, as shown in Figure 1b. When the temperature was raised to 380 °C, a tiny amount of α-MoO_3_ (ca. 2%) could be detected. When heated to 440 °C, the mass fraction of α-MoO_3_ quickly increased to 97.7%. According to the five-parameter logistic function fitting the mass fraction (the red curve in Figure 1b), the transition from h-MoO_3_ to α-MoO_3_ begins at a temperature of 378.5 °C and ends at a temperature of 443.1 °C, assuming that 2% α-MoO_3_ can be detected by X-ray diffraction.

Figure 1b illustrates the relationship between the isotropic microstrain Δ*d*/*d* of h-MoO_3_ and the temperature. From 300 to 370 °C, the microstrain of h-MoO_3_ decreased from 1443.4 to 1147.0 (↓ 20.5%), indicating the improvement of the crystallinity of the h-MoO_3_ phase through annealing. When h-MoO_3_ transformed into α-MoO_3_ (i.e., the mass fraction increased from 2.2% to 86%), the corresponding microstrain increased sharply from 1147.0 to 2246.9 (↑ 95.9%), indicating that the h-MoO_3_→α-MoO_3_ transition generates a pronounced microstrain. The strong microstrain modifies the shape of MoO_3_ before and after the phase transition. Figure 1c represents the regular hexagonal prisms of the sample calcinated at 300 °C (h-MoO_3_). The prisms are hundreds of micrometres in length and tens of micrometres in diameter, with a flat and smooth outer surface. In comparison, the hexagonal prisms in the sample calcinated at 400 °C (α-MoO_3_) are subdivided into numerous tabular microstructures, as displayed in Figure 1d. The tabular microstructures have a thickness of about 100 nm and a width of a few micrometres. The anomalous increase of the microstrain during the h-MoO_3_→α-MoO_3_ transition is determined by the local atomic coordination environments in the h-MoO_3_ structure, which is discussed in detail in Section “The structural phase transition from h-MoO_3_ to α-MoO_3_”.

Figure S1 in Supporting Information File 1 shows photodegradation experiments of methylene blue irradiated by ultraviolet light. The photodegradation rate of methylene blue was quantitatively estimated by pseudo-first-order kinetics. The reaction rate constants (*k*) are 0.01625, 0.01882, and 0.01258 min^−1^ for the samples calcinated at 300, 430, and 450 °C, respectively. The morphologies of the three samples (the inset of Figure S1 in Supporting Information File 1) are, respectively, smooth hexagonal prisms in h-MoO_3_, phase boundaries between h-MoO_3_ and α-MoO_3_, and numerous tabular microstructures in α-MoO_3_, implying that the h/α phase boundary may improve the photocatalytic performance of MoO_3_.

### The crystal structures of h-MoO_3_ and α-MoO_3_

#### The crystal structure of the hexagonal phase h-MoO_3_

Thermogravimetric results [23–25] indicate that the h-MoO_3_ phase releases water molecules during heat treatment, suggesting that water molecules reside in the crystal structure of h-MoO_3_. To determine the crystal structures of h-MoO_3_ and α-MoO_3_, slowly scanned XRD patterns were acquired from the carefully ground powders calcinated at 375 °C and 450 °C, respectively.

**Figure 2:**
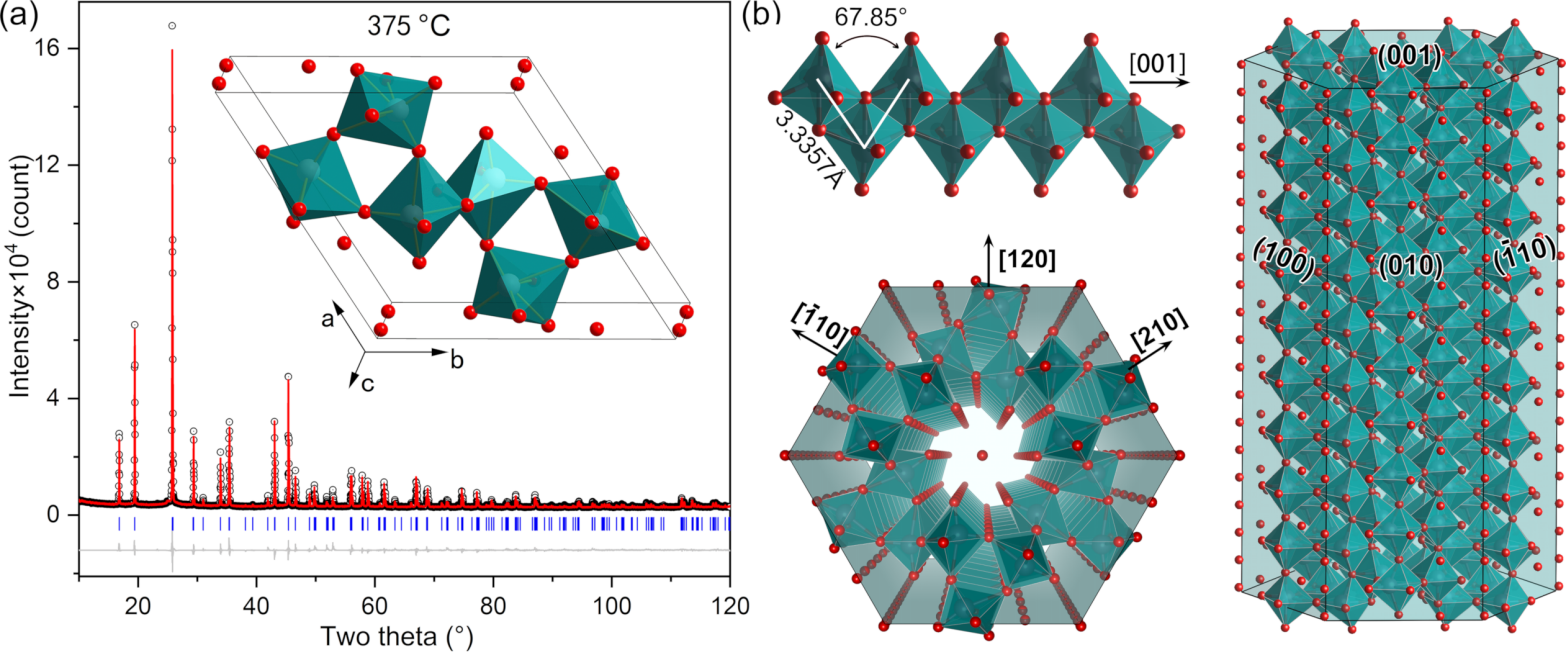
Crystal structure of the hexagonal phase MoO_3_·H_2_O from the sample calcinated at 375 °C. (a) Result of the Rietveld refinement; (b) illustrations of distorted MoO_6_ octahedra, an octahedra chain, and the hexagonal prism.

For the hexagonal phase h-MoO_3_, we performed Rietveld refinement based on two initial structural models. One model (MoO_3_·H_2_O) contains six water molecules, that is, the oxygen atom sites at (0 0 0.25) in the MoO_6_ octahedra tunnel (note that H atoms of the H_2_O molecules in the model were disregarded because of the low X-ray scattering ability). The other model (MoO_3_) does not contain any water molecules. The reliability factors of the former are 3.07%, 4.53%, 1.64%, and 5.43% for *R*_p_, *R*_wp_, *R*_exp_, and *R*_B_, respectively, slightly better than those of the latter (3.70%, 6.08%, 1.64%, and 7.95%, respectively), suggesting the presence of water molecules in the MoO_6_ octahedra tunnel of the hexagonal phase. Results of the Rietveld refinement are shown in Figure 2a, and the crystallographic data are listed in Table 1.

**Table 1:** The crystallographic data of the hexagonal phase refined on the MoO_3_·H_2_O and MoO_3_ models.

MoO_3_·H_2_O: *P*6_3_/*m* (61), *a* = 10.58185(13) Å, *c* = 3.72358(3) Å. *R*_p_ = 3.07%, *R*_wp_ = 4.53%, *R*_exp_ = 1.64%, and *R*_B_ = 5.43%.						MoO_3_: *P*6_3_/*m* (61), *a* = 10.58196(18) Å, *c* = 3.72359(4) Å. *R*_p_ = 3.70%, *R*_wp_ = 6.08%, *R*_exp_ = 1.64%, and *R*_B_ = 7.95%.					

Atom	Site	*x*	*y*	*z*	U_iso_ (Å^2^)	Atom	Site	*x*	*y*	*z*	U_iso_ (Å^2^)

Mo	6h	0.53865(15)	0.89289(4)	0.25	0.010	Mo	6h	0.53602(14)	0.89588(1)	0.25	0.010
O1	6h	0.56159(100)	0.50599(140)	0.25	0.015	O1	6h	0.56098(80)	0.50843(110)	0.25	0.015
O2	6h	0.52149(40)	0.72792(10)	0.25	0.015	O2	6h	0.50251(40)	0.72669	0.25	0.015
O3	6h	0.27882(120)	0.27442(90)	0.25	0.015	O3	6h	0.26911(80)	0.27992(90)	0.25	0.015
O4	6h	0	0	0.25	0.015						

In the structure of the hexagonal phase MoO_3_·H_2_O (Figure 2b), each Mo atom coordinates with six neighbouring O atoms to bond into a distorted MoO_6_ octahedron. Three nearest Mo atoms compose an isosceles triangle with 3.3357 Å for the sides and 3.7236 Å for the base so that the neighbouring MoO_6_ octahedra share edges to pack into a zigzag octahedra chain along the ⟨001⟩_h_ direction. Neighbouring octahedra chains share corners to form octahedra tunnels. Following the definition of the octahedra tunnel diameter by Lunk [24], the shortest O^…^O distance between diagonally located O atoms is 5.7053 Å. After subtracting the twofold van der Waals radius of O (1.52 Å), the diameter of the octahedra tunnel at 375 °C is 2.6653 Å, which is consistent with values given by Lunk (2.5–3.0 Å). In each unit cell, there are six water molecules intercalated inside the octahedra tunnel. The stacking of the neighbouring octahedra tunnels along the ⟨120⟩_h_ direction by sharing zigzag octahedra chains, together with the growth of octahedra chains along the ⟨001⟩_h_ direction stimulates MoO_3_·H_2_O to grow into the shape of hexagonal prisms seen in Figure 1b.

#### The crystal structure of the orthorhombic phase α-MoO_3_

**Figure 3:**
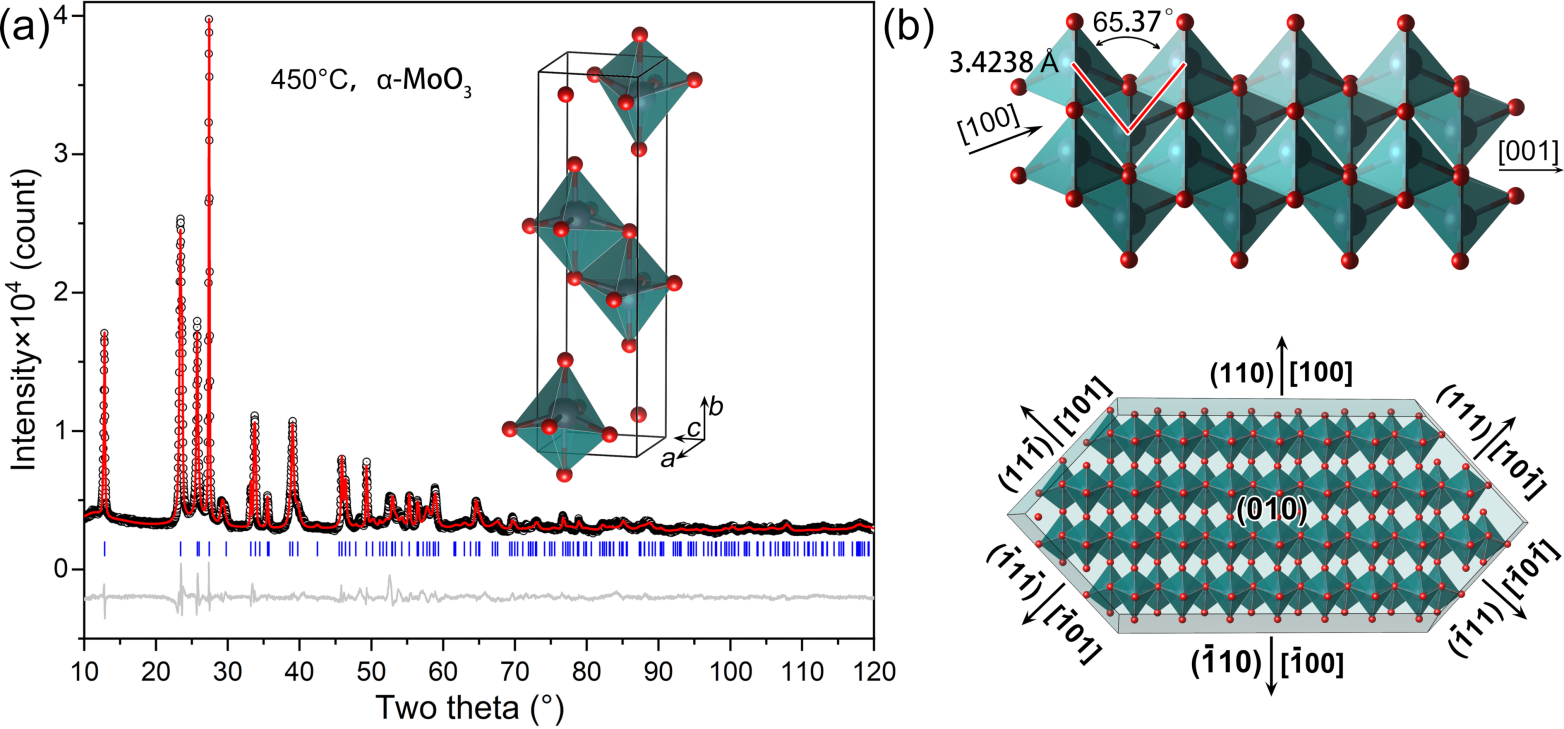
The crystal structure of the orthorhombic phase α-MoO_3_ from the sample calcinated at 450 °C. (a) Result of the Rietveld refinement; (b) illustrations of distorted MoO_6_ octahedra forming an octahedron layer and crystalizing into the tabular structure.

Figure 3a represents the Rietveld refinement of the orthorhombic phase α-MoO_3_ from the sample calcinated at 450 °C. The reliability factors *R*_p_, *R*_wp_, *R*_exp_, and *R*_B_ are 3.22%, 4.54%, 1.66%, and 4.01%, respectively. Table 2 lists the crystallographic data of α-MoO_3_. In the orthorhombic phase α-MoO_3_, the Mo atoms coordinate with the neighbouring O atoms to bond into distorted MoO_6_ octahedra. As seen in Figure 3b, neighbouring octahedra share edges to form a MoO_6_ octahedra chain in the ⟨001⟩_α_ direction, and the neighbouring chains share corners to form an octahedron layer along the ⟨100⟩_α_ direction. The octahedron layer grows along the ⟨100⟩_α_, ⟨101⟩_α_, and ⟨010⟩_α_ directions to crystalize into the tabular structure seen in the SEM experiment (Figure 1d).

**Table 2:** Crystallographic data of the orthorhombic phase α-MoO_3_ extracted from the sample calcinated at 450 °C. The Rietveld factors are *R*_p_ = 3.22%, *R*_wp_ = 4.54%, *R*_exp_ = 1.66%, and *R*_B_ = 4.01%.

α-MoO_3_: *Pnma* (62), *a* = 3.95825(18) Å, *b* = 13.8640(8) Å, *c* = 3.69801(14) Å, *V* = 202.937(25) Å^3^.					

Atom	Wyck.	*x*	*y*	*z*	*U*_iso_ (Å^2^)

Mo	6h	0.0617(7)	0.10242(21)	0.25	0.010
O1	6h	0.417(6)	0.4397(9)	0.25	0.015
O2	6h	0.5567(30)	0.0925(10)	0.25	0.015
O3	6h	0.100(5)	0.2394(10)	0.25	0.015

### The structural phase transition from h-MoO_3_ to α-MoO_3_

#### Anisotropic thermal expansion and first-order phase transition

**Figure 4:**
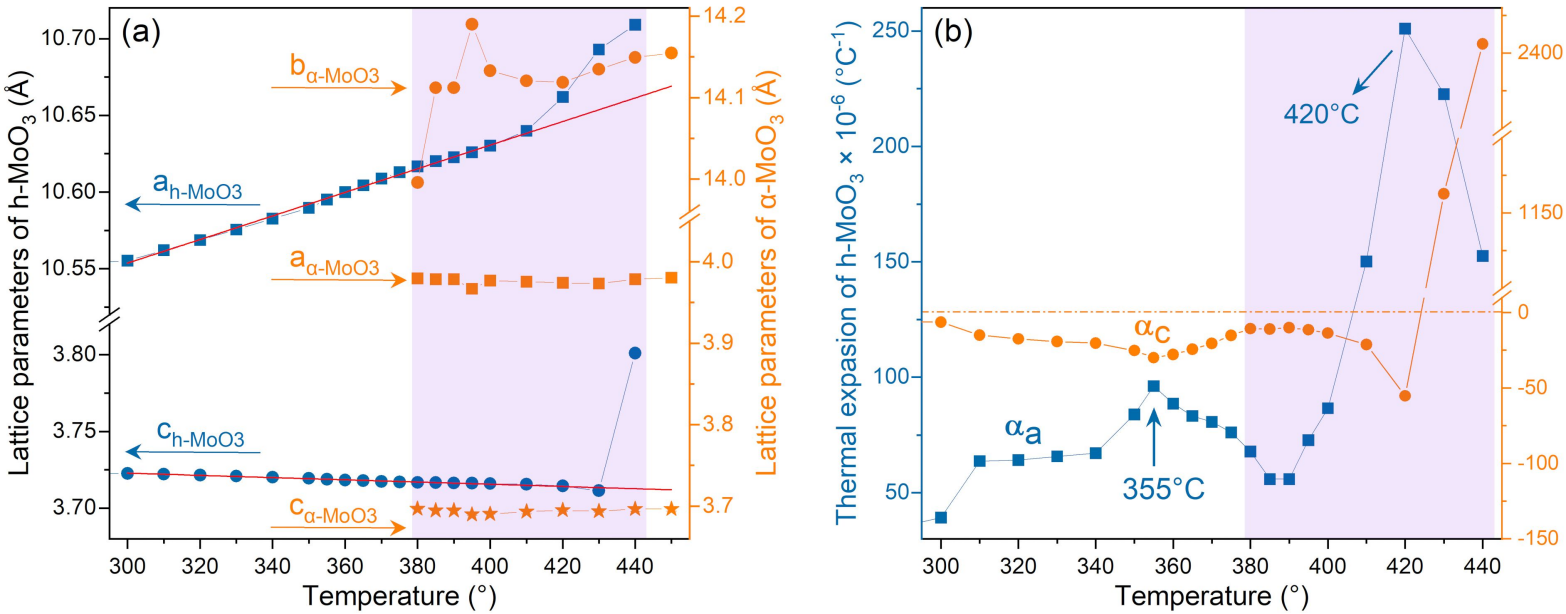
(a) Lattice parameters and (b) coefficients of thermal expansion as functions of the temperature during the transition from the hexagonal phase h-MoO_3_ to the orthorhombic phase α-MoO_3_. The red lines are the linear fits of *a* axis and *c* axis.

The coefficient of thermal expansion (CTE) is an important mechanical parameter for the application of MoO_3_ thin films, as it can increase the mismatch between thin films and the substrate, causing microdevices to be deformed or damaged. To estimate the CTE of the hexagonal phase h-MoO_3_, lattice parameters as a function of temperature were obtained from the Rietveld refinement of the in situ XRD patterns, as shown in Figure 4a. When the temperature was raised from 300 to 400 °C, the lattice parameter *a* and the unit cell volume increased roughly linearly with slopes of 769.14 × 10^−6^ and 0.04558, respectively, while the lattice parameter *c* decreased linearly with a slope of −72.37 × 10^−6^. The CTE can be calculated using the formula α*_x_* = (1/*x*)(d*x*/d*T*), where *x* represents the lattice parameters *a*, *b*, *c*, or the unit cell volume *V*. Thus, we determined the values of linear CTE and bulk CTE of the hexagonal phase h-MoO_3_ to be α*_a_* = α*_b_* = 72.87 × 10^−6^, α*_c_* = −19.44 × 10^−6^, and α*_V_* = 126.91 × 10^−6^ K^−1^, respectively, indicating that the hexagonal phase h-MoO_3_ has strongly anisotropic CTEs. Here, we compare the determined CTE values of h-MoO_3_ with those of α-MoO_3_ [26], α*_a_* = 6.66–10.03, α*_b_* = 32.94–45.64, α*_c_* = (−1.77)–(−1.86), and α*_V_* = (12.61–17.99) × 10^−6^ K^−1^. Remarkably, α*_a_* and α*_c_* of the hexagonal phase h-MoO_3_ are significantly larger, that is 7–10 times higher than those of α-MoO_3_.

In Figure 4b, the peak in α*_a_* at 355 °C is related to the release of the water molecules intercalated in the octahedra tunnel, which is consistent with the thermal analysis [23,27]. When increasing the temperature above 400 °C, the relation between lattice parameters and temperature evidently deviates from linearity. The parameter *a* increases from 10.63031 to 10.709 Å, resulting in a sharp increase of the CTE (or the first-order derivative of lattice parameters). This feature indicates that the h-MoO_3_→α-MoO_3_ transition is a first-order structural phase transition. The sharp increase of the thermal expansion α*_a_* causes an anomalous increase of microstrain during the h-MoO_3_→α-MoO_3_ transition.

Note that since the thermal shock properties of a material are proportional to the CTEs, large values of CTE and the anisotropy of the hexagonal phase h-MoO_3_ may increase thermal shock, leading to fatigue or damage to devices.

#### Microscopic view on the phase transition from h-MoO_3_ to α-MoO_3_

**Figure 5:**
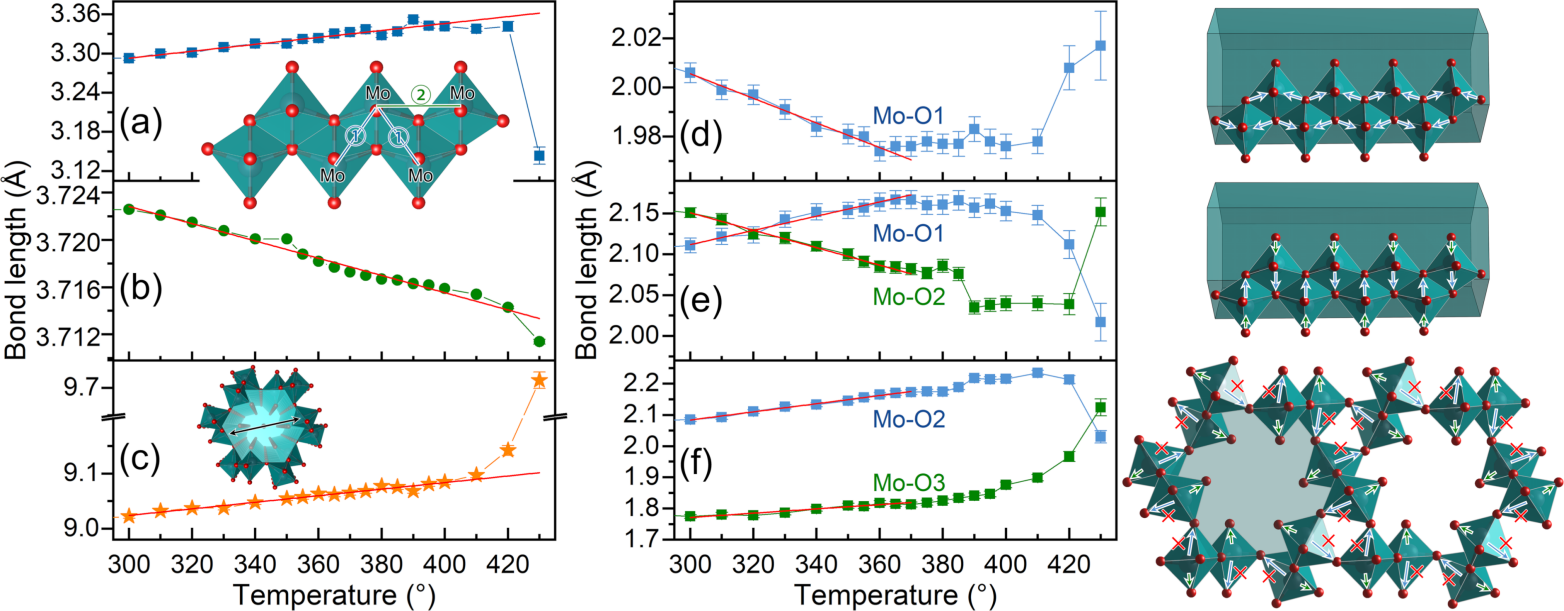
Variation of the local atomic bonding environment of h-MoO_3_ with temperature. (a,b) Lengths of the Mo–Mo bonds marked (1) and (2) in the inset. (c) Diameter of the octahedra tunnel as a function of the temperature. (d–f) Lengths of the Mo–O bonds within the octahedra corresponding to the given structures.

From the microscopic point of view, the variation of the lattice parameters depends on the local stacking feature of MoO_6_ octahedra in the h-MoO_3_ crystal structure. Figure 5a–c depicts the relationship between the neighbouring Mo–Mo bond lengths and the temperature. With the increase of temperature from 300 to 400 °C, the nearest-neighbour Mo–Mo bond (1) in Figure 5a increased roughly linearly from 3.2929 to 3.3419 Å (↑ 1.49%), while the next-neighbour Mo–Mo bond (2) in Figure 5b decreased roughly linearly from 3.7226 to 3.7159 Å (↓ 0.18%). These two features indicate the contraction of the zigzag octahedra chain along the axial ⟨001⟩_h_ direction.

The distinctive feature of the hexagonal phase h-MoO_3_ are the MoO_6_ octahedra tunnels, in which small molecules can be intercalated and which play a key role regarding the photophysical and photochemical properties [17,22]. Considering the correlation between the oxygen atomic position and the preferred orientation of the sample, here, we measured the diameter of the octahedra tunnel, which is approximately equivalent to the spacing of diagonally opposite Mo atoms, as illustrated in Figure 5c, rather than following H. J. Lunk's method. At 300 °C, the tunnel diameter is *D*_Mo–Mo_ = 9.0323 Å, equivalent to *D*_Lunk_ = 2.6192 Å in H. J. Lunk's method (*D*_Lunk_ ≈ *D*_Mo–Mo_ − 2*r*_0_ − 2*r*_eff_, where *r*_0_ = 1.52 Å is the van der Waals radius of O, and *r*_eff_ ≈ 1.6816 Å is the projection length of Mo–O2 along the measurement direction). From 300 to 400 °C, the tunnel diameter expanded linearly from 9.0225 to 9.0635 Å (↑ 0.45%), suggesting the release of the small molecules intercalated inside the tunnel. When the phase transition occurred, the tunnel diameter anomalously expanded to 9.1421 Å (↑ 1.33%) and 9.7137 Å (↑ 7.66%) at 420 and 430 °C, respectively, implying the collapse of the octahedra tunnel due to the dramatic increase of thermal expansion.

Changes in the Mo–Mo bonds are influenced by coordinated oxygen atoms that are sensitive to the thermal effect. Figure 5d–f displays the variation of the Mo–O bond lengths within the MoO_6_ octahedron as a function of the temperature. Along the octahedra chain direction, at 300 to 360 °C, the Mo–O1 bond length (Figure 5d) decreased approximately linearly from 2.006 to 1.974 Å (↓ 1.6%), and then remained nearly constant at this value up to 410 °C. This indicates that the decrease of the Mo–O1 bond length will pull the neighbouring octahedra closer (i.e., decrease the Mo–Mo (2) bond length), resulting in the contraction of the *c* axis of the unit cell and a negative α*_c_*. In the direction perpendicular to the octahedra chain (Figure 5e), at 300 to 360 °C, the Mo–O1 bond length increased linearly from 2.111 to 2.164 Å (↑ 2.51%), whereas the Mo–Mo2 bond length linearly dropped from 2.151 to 2.085 Å (↓ 3.07%). When heated to 420 °C, the Mo–O1 and Mo–O2 bonds evidently shortened, enhancing the strength of the octahedra chain in the direction perpendicular to the chain. Hence, the strength of the octahedra chain was enhanced in both the chain direction and the perpendicular direction.

In the radial direction of the octahedra tunnel (see Figure 5f), at 300 to 360 °C, the Mo–O2 bond length increased from 2.085 to 2.165 Å (↑ 3.84%) due to the release of water molecules, resulting in the expansion of the *a* axis of the unit cell and a peak in α*_a_*. When the temperature was raised to 410 °C, the Mo–O2 bond anomalously increased to 2.235 Å (↑ 7.19%), indicating significantly weakened or even broken Mo–O2 bonds (as denoted by the symbol “×” in Figure 5f). The breaking of the Mo–O2 bonds is also reflected by the relaxation of the Mo–O3 bond from a short bond (1.813 Å at 360 °C) to a normal value (1.967 Å at 400 °C).

**Figure 6:**
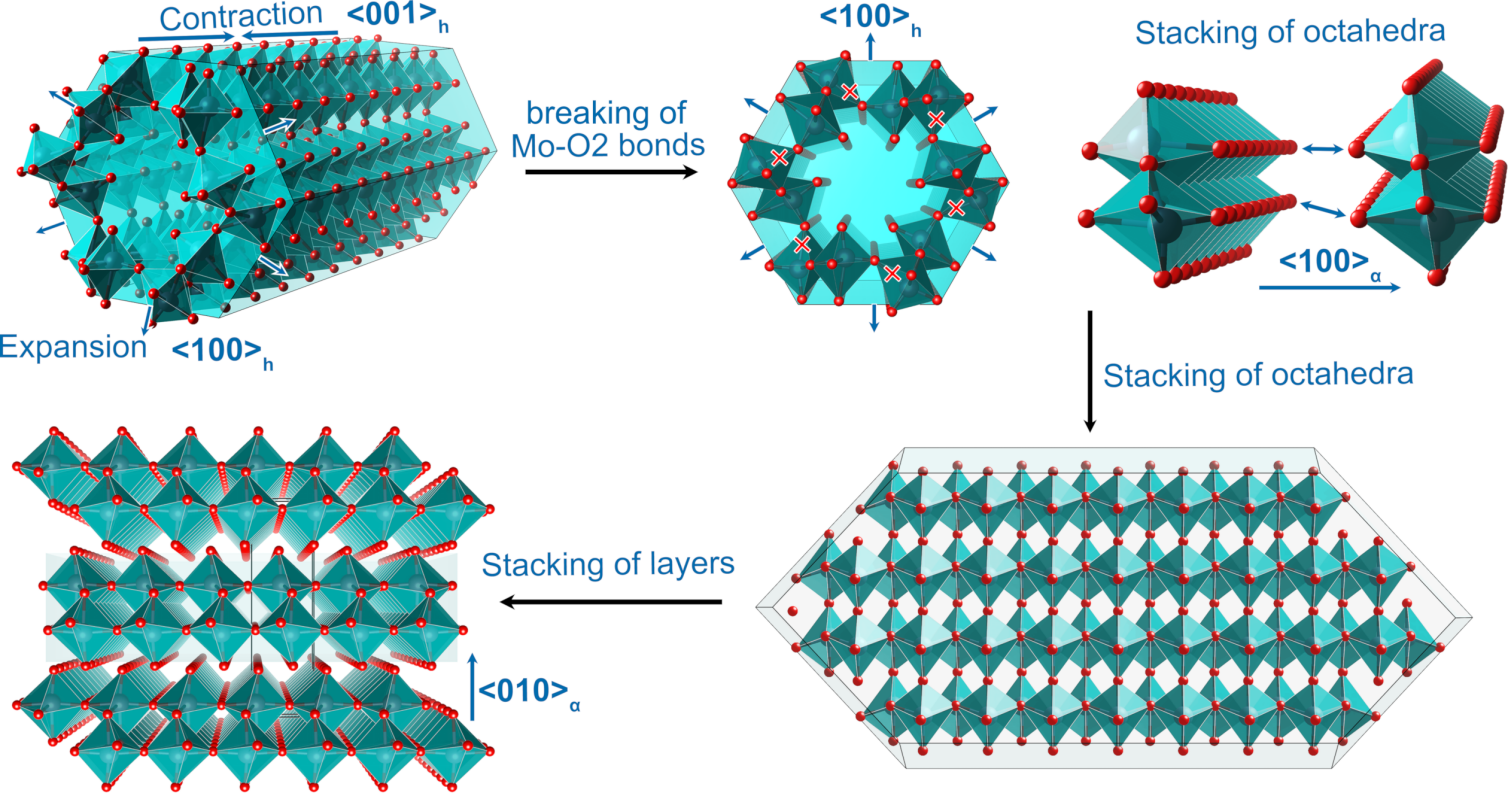
Phase transition from h-MoO_3_ to α-MoO_3_. Thermal expansion of the octahedra tunnel and the contraction of the octahedra chain, breaking of Mo–O2 bonds to destroy the octahedra tunnel into individual octahedra chains, stacking of octahedra chains to reconstruct the octahedron layers, and coupling of octahedron layers to form the α-MoO_3_ phase.

Based on the above analysis, we obtain the structural evolution of the h-MoO_3_→α-MoO_3_ phase transition, as illustrated in Figure 6. Heating the h-MoO_3_ phase causes not only an axial contraction of the octahedra chain but also the radial expansion of the octahedra tunnel, from which the intercalated molecules are released. When the phase transition occurs, the anomalous expansion of the octahedra tunnel ruptures the Mo–O2 bonds and divides the octahedra tunnel into MoO_6_ zigzag octahedra chains. The octahedra chains reconstruct into octahedron layers through corner sharing in the ⟨100⟩_α_ direction. The octahedron layers stack in the ⟨010⟩_α_ direction, coupled by van der Waals interactions, to form the α-MoO_3_ structure by shifting by **a**/2 between two neighbouring layers. As a result of the temperature-induced expansion of the MoO_6_ octahedra tunnel and the contraction of the octahedra chain, the microstrain increases dramatically, causing the hexagonal phase h-MoO_3_ to transform into the orthorhombic phase α-MoO_3_.

## Conclusion

Rietveld refinement indicates that the structure of the hexagonal phase h-MoO_3_ contains six water molecules intercalated in the octahedra tunnel in each unit cell. Before the phase transition, an increasing temperature induces the octahedra chain to contract and the octahedra tunnel to expand as water molecules are released from the tunnel, resulting in expansion of the *a* axis and contraction of the *c* axis of the unit cell. The values of linear CTE and the bulk CTE of h-MoO_3_ are α*_a_* = α*_b_* = 72.87 × 10^−6^ K^−1^, α*_c_* = −19.44 × 10^−6^ K^−1^, and α*_V_* = 126.91 × 10^−6^ K^−1^, respectively. These strongly anisotropic CTE values can increase thermal shock, which may destroy or damage MoO_3_ microdevices.

The h-MoO_3_→α-MoO_3_ transition is a first-order structure phase transition. When the phase transition occurs, the MoO_6_ octahedra tunnel anomalously expands, Mo–O2 bonds break, and individual MoO_6_ octahedra chains form. These chains share corners to generate octahedron layers in the ⟨100⟩_α_ direction, coupled by van der Waals interactions, and the octahedron layers stack in the ⟨010⟩_α_ direction by shifting by **a**/2.

## Experimental

### Hydrothermal synthesis of h-MoO_3_

Molybdenum oxide h-MoO_3_ was synthesized by a traditional hydrothermal synthesis method. In a routine procedure, 6.2 g of ammonium heptamolybdate was dissolved in 100 mL of deionized water and stirred for 30 min at room temperature. Nitric acid was then added to the solution to reduce the pH to 1. After stirring for another 15 min, the solution was transferred into a 200 mL Teflon-lined autoclave and heated at 120 °C for 12 h. The white precipitate was centrifuged (4000 rpm, 10 min) and washed with deionized water/ethanol three times. Finally, the precipitate was collected and dried at 70 °C for 6 h. The collected precipitate was divided into two parts. One was finely powdered and used as the sample for in situ X-ray diffraction experiments. The other was well mixed and ground and divided into eight parts that were calcined for 30 min at various temperatures (300–440 °C), and naturally cooled down to room temperature.

### Structure characterization

#### In situ X-ray diffraction

To investigate in situ the temperature-induced structural evolution of h-MoO_3_, finely ground powders were measured using an X-ray diffractometer (Rigaku SmartLab) equipped with a high-temperature holder HTTK600, using Cu Kα radiation (λ = 1.5406 Å). The 2θ range was 5–70°, and the temperature range was 30–450 °C. The scanning rate was 10 °·min^−1^, and the heating rate was 10 K·min^−1^. After reaching the target temperature, it was held for 5 min before collecting data.

#### X-ray diffraction of the calcinated samples

To solve and refine the crystal structure of the samples before and after the phase transition, samples calcinated at 375 and 450 °C were measured using the same X-ray diffractometer equipped with a standard holder. The 2θ range was 5–120°, and the scanning rate was 10 °·min^−1^.

#### Structure solution and Rietveld refinement

The crystal structure of the calcinated samples was solved using Jana2020 software [28]. The solved structures from calcinated samples as the initial models were refined using GSAS-II software [29]. In order to determine the temperature-induced structural evolution of h-MoO_3_, all in situ XRD patterns were refined in GSAS-II.

#### Microstructural characterization

Morphology and chemical composition of the calcinated samples were characterized using a field-emission scanning microscope (Hitachi S-4800) equipped with an energy-dispersive X-ray detector working at 10 kV and 10 μA.

## Supporting Information

File 1 Photocatalysis performance of the MoO_3_ samples.
