# Supplementary material for: The microstrain-accompanied structural phase transition from h-MoO3 to α-MoO3 investigated by in situ X-ray diffraction
Source: Beilstein J Nanotechnol. 2023 Jun 7;14:692–700. doi: 10.3762/bjnano.14.55 (PMC10280079; doi:10.3762/bjnano.14.55)
Supplement: File 1 — Photocatalysis performance of the MoO3 samples. [file Beilstein_J_Nanotechnol-14-692-s001.pdf]

BEILSTEIN JOURNAL OF NANOTECHNOLOGY

Supporting Information

for

The microstrain-accompanied structural phase transition from h-MoO_3 to α-MoO_3 investigated by in situ X-ray diffraction

Zeqian Zhang, Honglong Shi, Boxiang Zhuang, Minting Luo and Zhenfei Hu

Beilstein J. Nanotechnol. **2023,** *14,* 692–700. doi:10.3762/bjnano.14.55

Photocatalysis performance of the MoO_3 samples

License and Terms: This is a supporting information file under the terms of the Creative Commons Attribution License (https://creativecommons.org/licenses/by/4.0). Please note that the reuse, redistribution and reproduction in particular requires that the author(s) and source are credited and that individual graphics may be subject to special legal provisions.
The license is subject to the *Beilstein Journal of Nanotechnology* terms and conditions: (https://www.beilstein-journals.org/bjnano/terms)

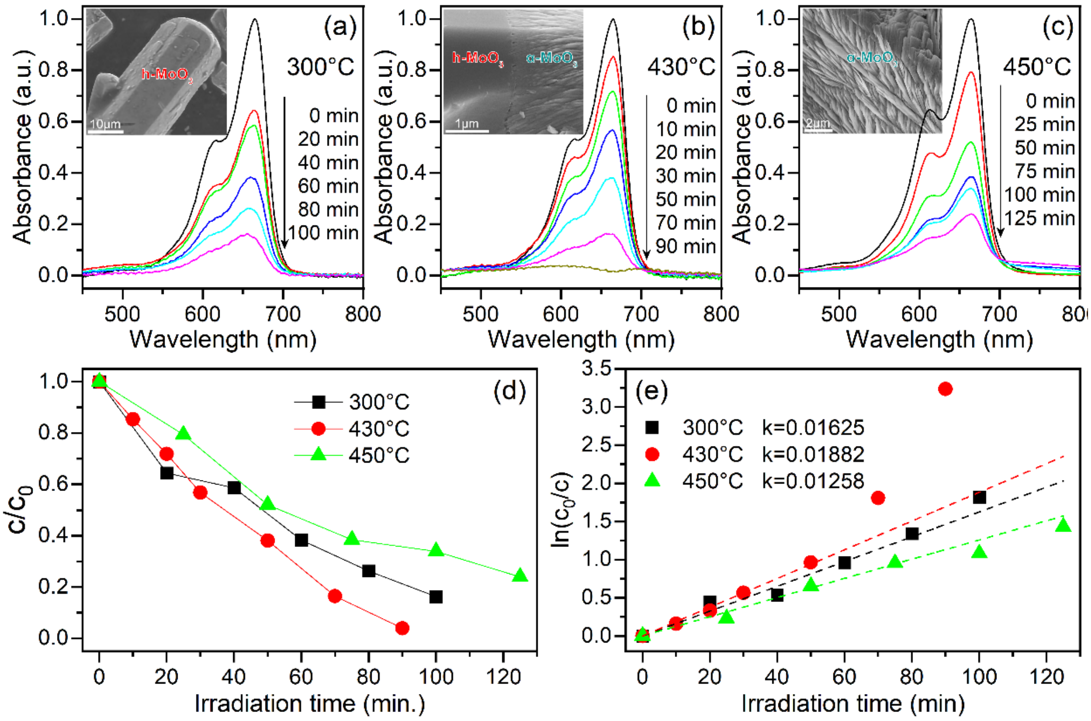

Figure S1: Photocatalysis performance of the MoO_3 samples. (a–c) UV–vis absorption spectra of methylene blue mixed with samples calcinated at 300, 430, and 450 °C. The insets display SEM micrographs of the samples. (d) Photodegradation c/c_0, and (e) $\ln(c_0/c)$ as a function of the irradiation time. The dotted lines are linear fits.
